# Promising Mid-Term Outcomes after Humeral Head Preserving Surgery of Posterior Fracture Dislocations of the Proximal Humerus

**DOI:** 10.3390/jcm10173841

**Published:** 2021-08-27

**Authors:** Lukas F. Heilmann, J. Christoph Katthagen, Michael J. Raschke, Benedikt Schliemann, Helmut Lill, Hassan El Bajjati, Gunnar Jensen, Rony-Orijit Dey Hazra

**Affiliations:** 1 Department of Trauma, Hand and Reconstructive Surgery, University Hospital Münster, 48149 Münster, Germany; christoph.katthagen@ukmuenster.de (J.C.K.); michael.raschke@ukmuenster.de (M.J.R.); benedikt.schliemann@ukmuenster.de (B.S.); 2 Department of Orthopedic and Trauma Surgery, Diakovere Friederikenstift and Henriettenstift, 30169 Hannover, Germany; helmut.lill@diakovere.de (H.L.); hassan.elbajjati@diakovere.de (H.E.B.); gunnar.jensen@diakovere.de (G.J.); rony-orijit.deyhazra@diakovere.de (R.-O.D.H.)

**Keywords:** proximal humerus fracture, posterior shoulder dislocation, humeral head preserving therapy, surgical neck fractures

## Abstract

Background: The aim of this study was to evaluate the clinical outcome after humeral head preserving surgical treatment of posterior fracture dislocations of the proximal humerus. Methods: Patients with a posterior fracture dislocation of the proximal humerus that were operatively treated in two level-1 trauma centers within a timeframe of 8 years were identified. With a minimum follow-up of 2 years, patients with humeral head preserving surgical treatment were invited for examination. Results: 19/24 fractures (79.2%; mean age 43 years) were examined with a mean follow-up of 4.1 ± 2.1 years. Of these, 12 fractures were categorized as posteriorly dislocated impression type fractures, and 7 fractures as posteriorly dislocated surgical neck fractures. Most impression type fractures were treated by open reduction, allo- or autograft impaction and screw fixation (*n* = 11), while most surgical neck fractures were treated with locked plating (*n* = 6). Patients with impression type fractures showed significantly better ASES scores (*p* = 0.041), Simple Shoulder Test scores (*p* = 0.003), Rowe scores (*p* = 0.013) and WOSI scores (*p* = 0.023), when compared to posteriorly dislocated surgical neck fractures. Range of motion was good to excellent for both groups with no significant difference. Conclusions: This mid-term follow-up study reports good to very good clinical results for humeral head preserving treatment.

## 1. Introduction

Proximal humeral fractures account for 4–5% of all fractures [1]. The age distribution is bimodal, with fragility fractures being more common in the elderly, while high-velocity trauma fractures tend to occur in younger patients [1,2]. Severely displaced fracture patterns of the humeral head are especially common in the latter patient group [3]. Among those, locked dislocated fractures of the proximal humerus are rare, as previous studies were able to show [4,5]. Within this cohort of locked dislocation fractures of the humerus, there are generally two fracture sub-groups concerning the direction of dislocation: the more common anterior dislocation of the fractured humeral head (AFD), which accounts for over 90% of cases, and the rarer posterior fracture dislocation (PFD) [3,6]. Studies reported that PFDs only account for approximately 0.9% of all shoulder fractures and affect 0.6/100.000 of the population per year [5,7]. Rouleau et al. compiled a systematic review that demonstrated that a majority of shoulder dislocations (65%) had associated injuries, with fractures being the most common, followed by reverse Hill-Sachs lesions (RHSL) and rotator cuff tears [8]. The range of surgical possibilities emphasizes the complexity of this injury [8]. High-velocity trauma and epileptic seizures have been reported as major causes of PFDs [9,10,11].

The heterogeneity in different fracture morphologies leads to various treatment options consisting of conservative therapy after closed reduction and several different surgical approaches. Operative approaches include open reduction and internal fixation, defect-filling (allo- and/or autograft; mod. McLaughlin procedure), hemi-arthroplasty, and primary (reverse) total arthroplasty [8,9,10,11]. Because of the rare incidence and the broad spectrum of therapeutic approaches regarding PFD, there is a small number of clinical studies in the literature, mainly focusing on primary arthroplasty [6,12], and while those studies report acceptable to good outcome parameters with hemi-arthroplasty and primary reverse arthroplasty, there is a lack of clinical outcome studies focusing on treatments that would preserve the humeral head instead of replacing it.

Therefore, the aim of the present study was to evaluate the mid-term clinical outcome of operatively treated PFDs with a focus on humeral head preserving (HHP) techniques. The hypothesis was that HHP surgical treatment leads to a good clinical mid-term outcome and a low complication rate.

## 2. Materials and Methods

### 2.1. Selection Criteria

This study has been performed in accordance with the ethical standards in the 1964 Declaration of Helsinki and after local ethical board approval (IRB No. AZ 2019-063-f-S). Within eight years (January 2010–January 2018), operatively treated patients with a PFD of the proximal humerus were identified in two level-1 trauma centers with specialized shoulder units. In this search, all patients that were at least 18 years of age at the time of trauma were included (*n* = 41).

The PFDs were subdivided by fracture morphology in:

- Impression fractures of the humeral head (29%)
- (Multifragmentary) surgical neck fractures (18.5%)
- Fractures of the lesser tuberosity (14.3%)
- Fractures of the greater tuberosity (7.8%)
- Other fractures (6%)

Exclusion criteria were defined as follows:

- Previous injuries or surgery to the affected shoulder
- Immunosuppressive therapy
- Drug or alcohol abuse
- Patients treated more than three weeks after trauma
- Patients that were treated with arthroplasty (*n* = 11)
- Patients that had died by the time the follow-up was conducted (*n* = 6)

### 2.2. Retrospective Analysis

Twenty-four fractures were identified that fit the selection criteria. Age, gender, and dates of trauma and surgery were recorded. Pre-, intra-, and postoperative X-ray images were analyzed. All patients underwent a pre-operative CT scan with 3D-reconstruction prior to surgery.

Two groups were created according to their fracture morphology: (1) impression type fractures (ITFs), also known as reverse Hill-Sachs lesions (RHSL) or Malgaigne fractures, that presented with an impression type defect of the proximal humerus but no fracture line at the surgical neck and (2) posteriorly displaced surgical neck fractures of the proximal humerus (SNFs) that presented with at least one fracture line at the surgical neck. The SNF group was classified according to the Resch, Codman, and Neer classification systems [13,14,15]. To further assess the extent of the defects within the ITF group, the gamma angles were assessed, as described by Moroder et al. [16]. Surgical treatment methods were recorded, and post-operative complications were determined.

### 2.3. Follow-Up Examination

A standardized follow-up exam took place 6 weeks postoperatively to check for fracture consolidation. Afterwards, patients would conduct follow-up exams with their respective primary physicians in the ambulatory sector.

The follow-up exam for this study was conducted in the clinic a minimum of 2 years postoperatively. The first part of the follow-up consisted of questions covering general data, such as pain level, patient satisfaction with the outcome, analgesia, and an assessment of the return to work/sport. The last X-ray in a.p. and y-view projection was analyzed for fracture consolidation or possible complications, and the head-shaft angle was calculated.

Next was an evaluation of the range of motion and clinical tests, including the age- and gender-adjusted Constant Score (CS), Simple Shoulder Test (SST), Subjective Shoulder Value (SSV), Rowe Score, WOSI Score, and ASES Score [17].

The Constant Score (CS) is a 100-point scale (high scores = high level of function) composed of individual parameters looking at pain, activities of daily living (ADL), mobility, and measuring the strength of the affected shoulder.

The Simple Shoulder Test (SST) is a questionnaire of 12 items focusing on pain, mobility, and strength.

The Subjective Shoulder Value (SSV) asks the patient for his/her assessment of the shoulder performance.

The Rowe Score is a 100-point scale (high scores = high level of function) with 50 points for stability, 20 points for range of motion, and 30 points for function.

The Western Ontario Shoulder Instability Index (WOSI) Score consists of 21 questions. The questions are scored from 0 to 100 using a visual analog scale. The overall scores range from 0 to 2100 (low scores = high level of function).

The American Shoulder and Elbow Surgeons (ASES) Score focuses on pain (7 items) and on ADL (10 items). Overall scores range from 0 to 100 (high scores = high level of function).

For the range of motion values, we defined the following cut-offs based on the values of the CS (Table 1) [18]:

### 2.4. Surgical Technique

The surgical technique varies based on different therapeutic approaches. In general, the senior surgeons of this article prefer a modified 30° beach chair positioning of the patient. As previously described, the fracture is exposed through a deltopectoral approach [19].

If an intraarticular reconstruction is necessary, a detachment or split of the subscapular muscle is performed for a direct visualization of the humeral head.

If possible, sutures are placed into the tendons of the supraspinatus and subscapularis muscle to reduce the greater and lesser tuberosity at the end of the surgery and prevent secondary displacement through pull of the muscle. In most cases, a soft tissue tenodesis of the long biceps tendon (LBS) is performed for further visualisation of the humeral head with the benefit of preventing postoperative tendinitis of the LBS. The rotator interval is opened, and an anatomical reduction of the fracture is performed.

In cases of a plate osteosynthesis, a lateral locking plate is applied and temporarily fixed while the reduction is verified through fluoroscopy. If reduction and plate positioning are satisfactory, all screws are inserted.

It may be advisable to augment by injecting bone cement into the apical screws of the humeral head if the bone quality is poor [20].

In cases with a lack of medial support or severe comminuted proximal humeral head fractures, an additional tubular plate is positioned at the lesser tuberosity. In external rotation of the humerus, the one-third tubular plate is placed anteriorly at the lesser tuberosity after moulding it to the correct shape, at a 30–45° angle to the lateral plate. A well-fitting plate prevents subcoracoid impingement. The correct position is achieved when the plate starts proximally at the lesser tuberosity at the insertion of the subscapular muscle, crosses the sulcus intertubercularis tendon, and ends laterally under the pectoralis major muscle.

**Figure 1:**
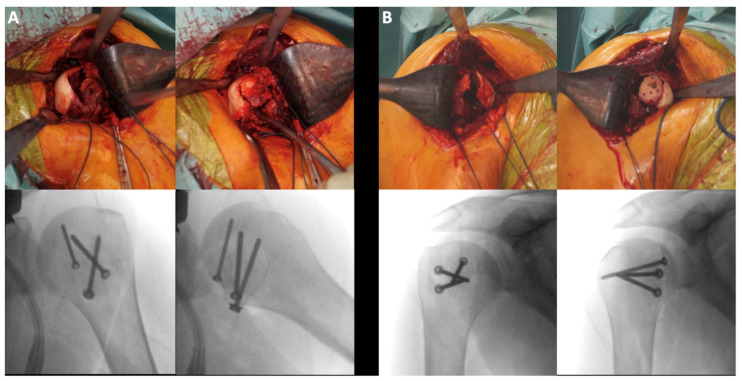
33-year old male patient after an epileptic seizure: bilateral posteriorly dislocated impression type fractures of the proximal humerus; (**A**) intraoperative pictures and X-rays of the left proximal humerus: open reduction, allo- and autograft, fixation with three screws (3.5 mm + 2.7 mm); (**B**) intraoperative pictures and X-rays of the right proximal humerus: open reduction, allo- and autograft, fixation with three screws (3.5 mm + 2.7 mm).

The initial steps of the surgical technique for ITFs are similar to the technique described above. After visualization of the fracture, the impression type fragment is reduced. In most cases, an open reduction and internal fixation with 2.7 and/or 3.5 mm cortical screws was performed with additional defect-filling (auto- and/or allograft) (Figure 1). This surgical method is similar to the one used for proximal tibia fractures [18]. For an autograft, bone material from the proximal humerus itself was used, or if a larger amount was necessary, a bone block from the ipsilateral iliac crest was used. For allograft, bone chips were used (Tutoplast (RTI surgical, Marquette, MI, USA)). Some fractures were treated by reduction and auto-/allograft alone without osteosynthesis.

### 2.5. Statistical Analysis

The data analysis was performed with GraphPad Prism (GraphPad 9.0.0, San Diego, CA, USA). The results were compared with Mann‒Whitney tests (two-sample unpaired *t*-tests) with a correction for multiple comparison using the Holm‒Šídák method. The level of significance was set to *p* < 0.05. All results displayed represent mean ± SD unless stated otherwise.

## 3. Results

Out of the 24 fractures that matched inclusion criteria, follow-up data was collected for 19 fractures (79.2%; Table 2).

**Figure 2:**
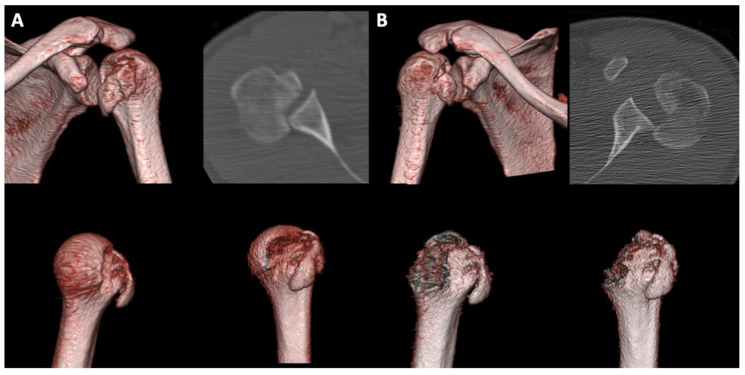
Preoperative imaging of patient from Figure 1; (**A**) preoperative axial CT-scan and 3D-VRT of the left glenohumeral joint; (**B**) preoperative axial CT-scan and 3D-VRT of the right glenohumeral joint.

Twelve fractures were subcategorized as ITFs and seven fractures as SNFs. Two patients had bilateral fractures, so we were able to follow-up with 17 patients (77.2%), representing 19 fractures. The mean age for these 19 cases was 43 ± 11.4 years (range 29–68 years); 15× male, 4× female. While there was a difference in age among the two groups (ITF mean 37 ± 9 years, SNF mean 52 ± 10 years), that difference was statistically not significant. The most common cause of injury (*n* = 11) was a fall or high energy trauma with the remaining eight cases caused by a seizure or by accidental electrocution (Figure 2). On average, these patients were treated within 6 ± 6.3 days after trauma (range 0–21 days). In all cases, a CT scan was performed before surgery. None of the fractures showed a concomitant glenoid fracture, and no injury of blood vessels or nerve injury associated with the fracture was observed.

### 3.1. Impression Type Fractures (ITFs)

**Figure 3:**
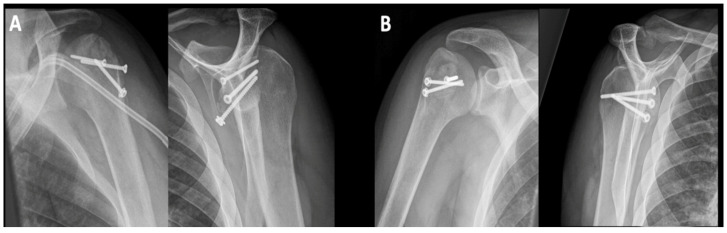
Postoperative imaging of the patient from Figure 1 and Figure 2; (**A**) left shoulder: 3-month postoperative X-rays of the left proximal humerus: no pain, no displacement of the screws (the opacification on the a.p. X-ray is a dialysis catheter); (**B**) right shoulder: 3-month postoperative X-rays of the left proximal humerus: no pain, no displacement of the screws.

The mean age for the ITF group was 37 ± 9 years. Eight out of twelve ITFs were still dislocated during the examination and were treated initially by closed reduction and immobilization in a sling in external rotation until surgery. Two ITFs were not dislocated at the inspection and were immobilized in a sling. The remaining two ITFs were still dislocated, but the closed reduction was unsuccessful. The mean gamma angle of the ITF defects was 101.4° ± 11°. Six of the twelve fractures were treated by open reduction and internal fixation with a screw with additional auto-/allograft. One fracture was treated with plate fixation, one fracture was addressed using a modified McLaughlin procedure and screw fixation, and four fractures were treated with auto- and allograft alone. Overall, in ten out of twelve ITFs, auto- and/or allograft was used to address the bone loss caused by the ITF. The average surgery time was 88 min (33–142 min) with all surgeries being performed by experienced shoulder surgeons. In one case, implant removal and arthroscopic arthrolysis of the shoulder joint were performed. No further complications were observed over time (Figure 3). All patients were able to return to work and their pre-injury level of sports.

### 3.2. Surgical Neck Fractures of the Humerus (SNFs)

For the SNF group (*n* = 7), the mean age was 52 ± 10 years. Six out of seven SNFs were classified as Resch 5GL, Codman 4-part, and Neer type IV fractures (4-part). The remaining fracture was classified as a Resch 5G, Codman 3-part, and Neer type IV fracture (3-part). All SNFs presented with an ITF component in addition to the SNF with a mean gamma angle of 108.2° ± 11° for the bone defect. Furthermore, five out of seven fractures showed head-split fractures on the CT-scan.

Six of the seven fractures were treated with locked plate fixation via a deltopectoral approach, and one fracture was fixed with screws only. In more than half of the cases (four out of seven), the surgeons opted for a double plate fixation (DPO), in which an anterior one-third tubular plate was used in addition to the lateral locking plate. Furthermore, in four out of seven cases, auto- or allograft was used to treat bone defects. The average surgery time was 135 min (70–202 min) with all surgeries being performed by experienced shoulder surgeons.

### 3.3. Revision Surgery & Complications

**Figure 4:**
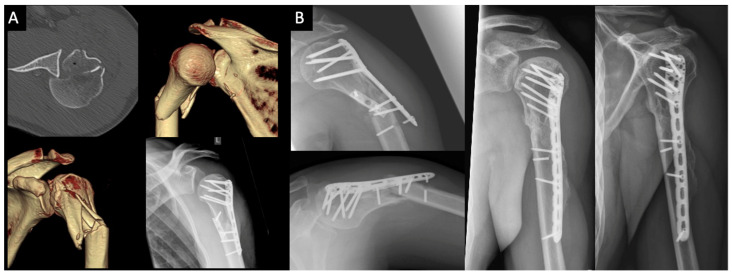
39-year old male bicyclist that collided with a motor vehicle; (**A**) axial CT scan and 3D reconstruction of a posteriorly dislocated surgical neck fracture with an additional reverse Hill-Sachs lesion; (**B**) secondary dislocation with screw failure (left) and final result after surgical revision 5.5 years after the index injury (right).

Figure 4 shows the case of a 39-year-old male bicyclist that collided with a motor vehicle. He presented with a posteriorly dislocated locked SNF and an additional RHSL. The surgeons treated this case via double-plate fixation (Figure 3A). Unfortunately, this patient was re-admitted to the clinic 6.5 months postoperatively with implant failure and secondary dislocation of the fracture (Figure 3B, left) without de novo trauma. Consequently, an implant removal was performed, followed by corrective osteotomy, bone autograft, and re-fixation with a longer plate (Figure 3B, right). There were no further complications in the other SNF cases. Six out of seven patients were able to return to their work and their respective level of sports prior to the trauma.

Besides the one patient with implant failure, three patients (2× SNF group, 1× ITF group) underwent an implant removal with arthroscopic arthrolysis due to post-operative shoulder stiffness resulting in an overall complication rate of 21%.

### 3.4. Range of Motion and Functional Scores

The mean follow-up time was 4.1 ± 2.1 years (range 2–8.4 years) after surgery. All patients were satisfied with the results of the surgery. The mean range of motion was excellent for the ITF group and good for the SNF group (Table 1 and Table 3). The ITF group showed better values for forward flexion, abduction, and external rotation, even though those differences were not significant. Regarding the clinical tests, the ITF group achieved better scores for CS and SSV. This difference was also not statistically significant. For the remaining scores, namely SST, Rowe, WOSI, and ASES, statistically relevant differences could be observed in favor of the ITF over the SNF group (Table 3).

## 4. Discussion

The most important finding of this study is that humeral head preserving surgical treatment of posterior fracture dislocations of the proximal humerus yields a good clinical outcome. Secondly, patients with SNFs showed inferior outcomes compared to patients with ITFs [18,21,22].

### 4.1. Surgical Treatment Options

While most ITFs were treated via screw fixation and/or auto-/allograft, the choice of surgical treatment for SNFs looks much different. In almost all cases (6/7), locked plating was necessary to ensure a stable fixation after proper fracture reduction. In 5/7 cases, an auto- or allograft was also used. An additional graft was necessary because all SNFs also presented with bone defects (ITF/RHSL). These different approaches in surgical strategy underline the importance of understanding the pathology of these fractures. 

The results of this study mirror those in the literature of case series that show good to excellent results after open reduction surgery of SNFs [21,22,23]. Johnson and Pandey published a case series of 11 patients with three- and four-part SNFs and were able to show promising results (mean CS of 75 (range 64–86)) [22]. They treated all patients with a minimal open reduction and percutaneous screw fixation technique. Luigi et al. reported a case series of three SNFs with excellent results (CS range from 100–95 points) that were treated using locking plates [23]. While having a statistically lower CS compared to the ITFs, the SNF group in this study showed a good CS of 74 points. 

### 4.2. Clinical Outcome

Regarding the clinical outcome, we were able to report very good results for the ITF group (Table 3). All patients presented with a forward flexion and abduction of at least 155° and an external rotation of at least 60°. Furthermore, all patients in this group were able to return to their respective jobs and perform at their pre-injury level in sports. While the range of motion for the SNF group showed lower values, patients were still able to perform a mean forward flexion and abduction of at least 115° and a mean external rotation of 38°.

While the SNF group showed good results regarding the CS with 75 points, the ITF group presented very good results with a mean CS of 92 points. This difference was statistically not significant. This is in accordance with case reports and case series on this topic [3,21,24]. Trikha et al. analyzed the functional outcome of posterior fracture dislocations treated with locking plates in a level-4 study that included 33 patients [3]. Their patient group contained 27 (82%) cases of AFD and 6 (18%) cases of PFD with an overall CS of 78 in both groups. In another study, Oliveira et al. performed a follow-up on eight patients with posteriorly displaced SNFs treated with locking plates and reported good clinical outcomes (CS mean of 85 ± 13.6) [21].

Similarly, Gerber et al. were able to show a good long-term outcome (CS of 77 points (range, 52–98 points)) for 19 cases with ITFs treated with allo- and/or autograft as a segmental reconstruction of the humeral head with a mean post-operative follow-up time of 10.6 years [24]. The difference in CS values in this study among the groups was 18 points (92 vs. 74). In the literature, the claim has been made that a CS value of more than 6.7 points represents the minimally important clinical difference (MICD) in patients surgically treated with humeral shaft fractures [25]. Therefore, the detected difference in scoring can be considered to make a clinical difference for patients. Regarding the ASES Shoulder Score, the groups in this study showed a difference of 11 points (Table 3). This difference among groups was significant. The ITF group also showed significantly better scoring for the SST, Rowe, and WOSI score.

Besides the one case depicted above and the three cases of combined implant removal and arthrolysis, there were no other revision surgeries or complications observed during follow-up. This is in line with a large long-term follow-up study by Robinson et al. that evaluated complications and long-term outcomes of open reduction and plate fixation of proximal humeral fractures in 368 patients [26].

### 4.3. Engagement of Bone Defects

Regarding the recommendation for surgical treatment, Moroder et al. described a method to measure the critical value in a biomechanical study: the gamma angle [16]. This angle is a combination of size and localization of RHSL defects. Size and localization of the bone defect are both critical factors for engagement. The study group recommends surgical treatment for defects with a gamma angle greater than 90°. Our study measured mean gamma angles for the ITFs of 101.4° and mean gamma angles for those SNFs with an accompanying ITF of 108.2°.

### 4.4. Save the Humeral Head or Replace It?

As mentioned in the introduction, another option for ITFs as well as SNFs is hemi- or total arthroplasty. While the cut-off in defect size is still debated in current literature, some authors gave recommendations based on defect size [24,27]. Gerber et al. recommend treating ITFs with a defect size of the humeral joint surface of 35% to 40% or greater by hemi- or total arthroplasty, as reconstructions of such significant bone defects might compromise joint mobility [24]. Similarly, Schliemann et al. recommend treating RHSLs greater than 45% of the humeral joint surface with arthroplasty [27]. In line with those recommendations, all the patients that underwent humeral head preserving surgery included in this study showed a defect size of 35% or less.

### 4.5. Limitations

One of the limitations of this study is its small number in patient population. This is due to the scarcity of these fractures combined with a posterior dislocation of the proximal humerus. For future studies focusing on this fracture pattern, it would be necessary to include several level-1 trauma centers in a study to achieve higher numbers. Another limitation is the timing of the follow-up exam, as it was not standardized. This could be addressed in the future with a prospective clinical study.

## 5. Conclusions

This mid-term follow-up study reports good to very good clinical results for humeral head preserving treatment of posterior fracture dislocations of the proximal humerus with a mean four-year follow-up. This study advocates for treatment that preserves the humeral head, if clinically reasonable.

## Figures and Tables

**Table 1:** Rating system for the range of motion based on the Constant Score (CS).

Rating	Forward Flexion	Abduction
Excellent	151–180°	151–180°
Very good	121–150°	121–150°
Good	91–120°	91–120°
Fair	61–90°	61–90°
Bad	31–60°	31–60°
Very Bad	0–30°	0–30°

**Table 2:** Datasheet for fractures that were examined during follow-up showing age, gender, type of fracture, surgical treatment, and time of follow-up (TFU) in relation to surgery date in years; ITF = fracture, SNF = surgical neck fracture, SPO = single plate osteosynthesis, DPO = double plate osteosynthesis.

Pat. ID	Age	Gender	Fracture	Treatment	TFU (y) Follow-Up (y)
1	43	M	ITF	Allo- and autograft	7.6
2	31	W	ITF	Allograft	7.6
3	48	M	ITF	SPO, 1x screw	5.4
4	28	M	ITF	Allograft, 6x screw	2.4
5	33	M	ITF	Allo- and autograft, 3x screw	2.8
6	33	M	ITF	Allo- and autograft, 3x screw	2.8
7	27	W	ITF	Allograft	3.3
8	47	M	ITF	Allograft	3.4
9	47	M	ITF	Mod. McLaughlin procedure, 3x screw	4.6
10	29	M	ITF	Autograft, 2x screw	4.6
11	45	M	ITF	Allo- and autograft, 3x screw	4.3
12	35	M	ITF	Autograft, 2x screw	8.4
13	39	M	SNF	DPO	5.9
14	48	W	SNF	DPO	2.7
15	68	M	SNF	Allo- and autograft, 2x screw, SPO	2.2
16	47	M	SNF	2x screw, SPO	2.3
17	45	M	SNF	Autograft, 2x screw	4.2
18	60	W	SNF	SPO, 2x screw	2.5
19	58	M	SNF	Autograft, SPO, 1x screw	2.1

**Table 3:** Results of the functional outcome during follow-up: patients in the ITF group showed greater values than patients in the SNF group, with some significant differences (*). Mean column shows mean values, upper and lower limit shows upper and lower CI (95%); CS = Constant Score (corrected for age and gender); SST = Simple Shoulder Test; SSV = Subjective Shoulder Value; WOSI = Western Ontario Shoulder Instability Index; ASES = American Shoulder and Elbow Surgeons Standardized Shoulder Assessment Form.

	ITF (*n* = 12), Mean	SNF (*n* = 7), Mean	*p*-Value
Forward Flexion	172° (160–184°)	118° (65–172°)	0.421
Abduction	172° (160–184°)	115° (60–171°)	0.421
External Rotation	69° (60–77°)	38° (19–57°)	0.084
Head/shaft-angle	124.3° (121–127°)	120.9° (112–130°)	>0.999
CS	92 (84–99)	75 (55–93)	0.099
SST	100% (100–100%)	80% (53–100%)	0.003 *
SSV	91% (85–98%)	83% (68–98%)	0.421
Rowe	97 (92–100)	88 (78–97)	0.013 *
WOSI	11 (2–19)	34 (18–51)	0.023 *
ASES	96 (92–100)	85 (68–100)	0.041 *

## Data Availability

The data presented in this study may be available on request from the corresponding author. The data are not publicly available due to privacy and ethical considerations.

## References

[1] Passaretti D., Candela V., Sessa P., Gumina S. (2017). Epidemiology of proximal humeral fractures: A detailed survey of 711 patients in a metropolitan area. J. Shoulder Elb. Surg..

[2] Palvanen M., Kannus P., Niemi S., Parkkari J. (2006). Update in the epidemiology of proximal humeral fractures. Clin. Orthop. Relat. R.

[3] Trikha V., Singh V., Choudhury B., Das S. (2017). Retrospective analysis of proximal humeral fracture-dislocations managed with locked plates. J. Shoulder Elb. Surg..

[4] Alkaduhimi H., van der Linde J.A., Flipsen M., van Deurzen D.F.P., van den Bekerom M.P.J. (2016). A systematic and technical guide on how to reduce a shoulder dislocation. Turk. J. Emerg. Med..

[5] Robinson C.M., Akhtar A., Mitchell M., Beavis C. (2007). Complex posterior fracture-dislocation of the shoulder. J. Bone Jt. Surg..

[6] Kokkalis Z.T., Iliopoulos I.D., Antoniou G., Antoniadou T., Mavrogenis A.F., Panagiotopoulos E. (2017). Posterior shoulder fracture–dislocation: An update with treatment algorithm. Eur. J. Orthop. Surg. Traumatol..

[7] Neer C.S., Foster C.R. (1980). Inferior capsular shift for involuntary inferior and multidirectional instability of the shoulder. A preliminary report. J. Bone Jt. Surg..

[8] Rouleau D.M., Hebert-Davies J. (2012). Incidence of associated injury in posterior shoulder dislocation. J. Orthop. Trauma.

[9] Sandmann G.H., Siebenlist S., Imhoff F.B., Ahrens P., Neumaier M., Freude T., Biberthaler P. (2016). Balloon-guided inflation osteoplasty in the treatment of Hill-Sachs lesions of the humeral head: Case report of a new technique. Patient Saf. Surg..

[10] Yigit M., Yaman A., Yigit E., Turkdogan K.A. (2016). The overlooked side of convulsion: Bilateral posterior fracture and dislocation of proximal humerus. JPMA J. Pak. Med. Assoc..

[11] Heilmann L.F., Katthagen J.C., Raschke M.J., Lill H., Schliemann B., Bajjati H.E., Jensen G., Dey-Hazra R.O. (2019). Posterior fracture dislocation of the proximal humerus. Obere Extrem..

[12] Boyle M.J., Youn S.-M., Frampton C.M.A., Ball C.M. (2013). Functional outcomes of reverse shoulder arthroplasty compared with hemiar-throplasty for acute proximal humeral fractures. J. Shoulder Elb. Surg..

[13] Resch H., Tauber M., Neviaser R.J., Neviaser A.S., Majed A., Halsey T., Hirzinger C., Al-Yassari G., Zyto K., Moroder P. (2016). Classification of proximal humeral fractures based on a pathomorphologic analysis. J Shoulder Elb Surg..

[14] Neer C.S. (1971). Displaced fractures of the proximal humerus. Clin. Orthop. Relat. R.

[15] Guix J.M.M., Pedrós J.S., Serrano A.C. (2009). Updated classification system for proximal humeral fractures. Clin. Med. Res..

[16] Moroder P., Runer A., Kraemer M., Fierlbeck J., Niederberger A., Cotofana S., Vasvari I., Hettegger B., Tauber M., Hurschler C. (2015). Influence of defect size and localization on the engagement of reverse hill-sachs lesions. Am. J. Sports Med..

[17] Katolik L.I., Romeo A.A., Cole B.J., Verma N.N., Hayden J.K., Bach B.R. (2005). Normalization of the constant score. J. Shoulder Elb. Surg..

[18] Rodia F., Ventura A., Touloupakis G., Theodorakis E., Ceretti M. (2012). Missed posterior shoulder dislocation and McLaughlin lesion after an electrocution accident. Chin. J. Traumatol./Zhonghua Chuang Shang Za Zhi.

[19] Warnhoff M., Jensen G., Dey Hazra R.O., Theruvath P., Lill H., Ellwein A. (2021). Double plating—Surgical technique and good clinical results in complex and highly unstable proximal humeral fractures. Injury.

[20] Katthagen J.C., Lutz O., Voigt C., Lill H., Ellwein A. (2018). Cement augmentation of humeral head screws reduces early implant-related complications after locked plating of proximal humeral fractures. Obere Extrem..

[21] De Oliveira C.T.B., da Graça E., Fanelli V.A. (2018). Posterior four-part fracture-dislocations of the proximal humerus: Clinical and func-tional evaluation of osteosynthesis treatment. Rev. Bras. Ortop. Engl. Ed..

[22] Johnson N., Pandey R. (2018). Proximal humerus fracture–dislocation managed by mini-open reduction and percutaneous screw fixa-tion. Shoulder Elb..

[23] Luigi B.V., Stefano L., Mauro M. (2020). Locked posterior fracture-dislocation of the shoulder. Acta BioMed. Atenei Parm..

[24] Gerber C., Catanzaro S., Jundt-Ecker M., Farshad M. (2014). Long-term outcome of segmental reconstruction of the humeral head for the treatment of locked posterior dislocation of the shoulder. J. Shoulder Elb. Surg..

[25] Kukkonen J., Kauko T., Vahlberg T., Joukainen A., Äärimaa V. (2013). Investigating minimal clinically important difference for Constant score in patients undergoing rotator cuff surgery. J. Shoulder Elb. Surg..

[26] Robinson C.M., Stirling P.H.C., Goudie E.B., MacDonald D.J., Strelzow J.A. (2019). Complications and long-term outcomes of open re-duction and plate fixation of proximal humeral fractures. J. Bone Jt. Surg..

[27] Schliemann B., Muder D., Geßmann J., Schildhauer T.A., Seybold D. (2011). Locked posterior shoulder dislocation: Treatment options and clinical outcomes. Arch. Orthop. Trauma Surg..

